# Metabolic Regulation of Dendritic Cell Differentiation

**DOI:** 10.3389/fimmu.2019.00410

**Published:** 2019-03-13

**Authors:** Zhimin He, Xinyi Zhu, Zhen Shi, Tao Wu, Li Wu

**Affiliations:** ^1^School of Medicine, Institute for Immunology, Tsinghua University, Beijing, China; ^2^Tsinghua-Peking Center for Life Science, Beijing, China

**Keywords:** dendritic cell (DC), cell differentiation, metabolic regulation, glycolysis, fatty acid (FA), mitochondria function, mTOR pathway, nutrients

## Abstract

Dendritic cells (DCs) are important antigen-presenting cells (APCs) that play essential roles in bridging innate and adaptive immune responses. Differentiation stages of DC subsets from bone marrow progenitor cells have been well-defined during the past decades. Features that distinguish DC progenitor cells from each differentiation stages, related signaling pathways and transcription factors that are crucial for DC lineage commitment have been well-elucidated in numerous studies. Recently, growing evidence are showing that cellular metabolism, as one of the most fundamental process of cells, has essential role in the modulation of immune system. There have been multiple reports and reviews that focus on the metabolic modulations on DC functions, however little attention had been paid to the metabolic regulation of DC development and differentiation. In recent years, increasing evidence suggests that metabolic regulations also exert significant impact on DC differentiation, as well as on the homeostasis of tissue resident DCs. The focus of this review is to summarize the findings from recent studies on the metabolic regulation of DC differentiation and to discuss the impacts of the three major aspects of metabolism on the processes of DC development and differentiation, namely the changes in metabolic pathways, the molecular signaling pathways that modulate cell metabolism, and the effects of metabolites and nutrients. The aim of this review is to draw attentions to this important and exciting research field where the effects of metabolic process and their regulation in DC differentiation need to be further explored.

## Introduction

Dendritic cells (DCs) are specialized cells that not only recognize the pathogens by the various pattern recognition receptors (PRRs) and initiate the innate immune response, but also can uptake, process and present antigens to naïve T cells, thus promote the activation of adaptive immune response (1). Based on the expression of distinct cell surface molecules, the requirement for specific transcription factors essential for their development, the origins, and their tissue localizations, DCs can be classified into four types: the plasmacytoid DCs (pDCs), the conventional DCs (cDCs) which can be further divided into cDC1 and cDC2 subsets, the monocyte-derived DCs (moDCs), and Langerhans cells (LCs). DC subsets, especially cDCs acquire distinct features in different tissue environments. Characterizations of distinct functions of these DC subsets (2–4), and the molecular regulation network for their development and differentiation (5–7) have been well-summarized in many review articles. The major features of these DC subsets are summarized in Table 1 (7–11).

**Table 1:** Murine and human dendritic cell subsets are outlined with their surface phenotype, major transcription factors required for their development and their main functions (7–11).

		**Surface maker**					**Specific transcription factors**	**Common function**	
**DC subset**		**Murine**			**Human**			**Antigen presentation and cytokine production**	**Downstream effect**
cDC	cDC1	CD11c^hi^, MHC-II^+^, CD45RA^−^, Siglec H^−^, PCDA-1^−^	XCR1^+^, Clec9A^+^, DEC205^+^	CD8α^+^ (lymphoid tissues)	HLA-DR^+^, CD11c^+^, CD123^−^	XCR1^+^, CD141^+^, Clec9A^+^	Irf8, Id2, Batf3	Direct and cross-presentation IL-12, IL-6, Type III IFN	CD8^+^ T cell activation Th1 activation
				CD103^+^ (non-lymphoid tissues)					
	cDC2		Sirpα^+^, CD11b^+^	Esam^hi^, CD4^+^, Clec4a4^+^ (lymphoid tissues)		Sirpα^+^, CD1c^+^, CD301^+^	Irf4, Notch2, Klf4	Direct presentation IL-6, TNF-α IL-23 (intestinal and lung)	Th2 and Th17 activation
				Esam^lo^, Clec12A^+^, CD103^+/−^ (non-lymphoid tissues)					
pDCs		CD11c^int^, MHC-II^−^, CD45R^+^, CD45RA^+^, Siglec H^+^, PCDA-1^+^			CD11c^lo^, CD123^+^, CD45RA^+^, BDCA2^+^, BDCA4^+^		E2-2, Irf8, Bcl11a, Runx2, SpiB	Type I, Type III interferons IL-12, IL-6	Antiviral immunity
moDCs		CD11c^+^, MHC-II^+^, CD11b^+^			CD11c^+^, HLA-DR^+^, CD14^+^, BDCA1^+^, FcεRI^+^, CD206^+^		Klf4, Irf8	TNF-α, IL-12, IL-23, iNOS	Th1 and Th17 response
Langerhans cells		EpCAM^hi^, MHC-II^+^, CD11b^+^, CD11c^+^			CD11c^lo^, CD1a^hi^, Langerin^+^		Id2, Runx3	Dermal and epidermal antigen presentation IL-23, IL-6, IL-1β	Th17 response

**Figure 1:**
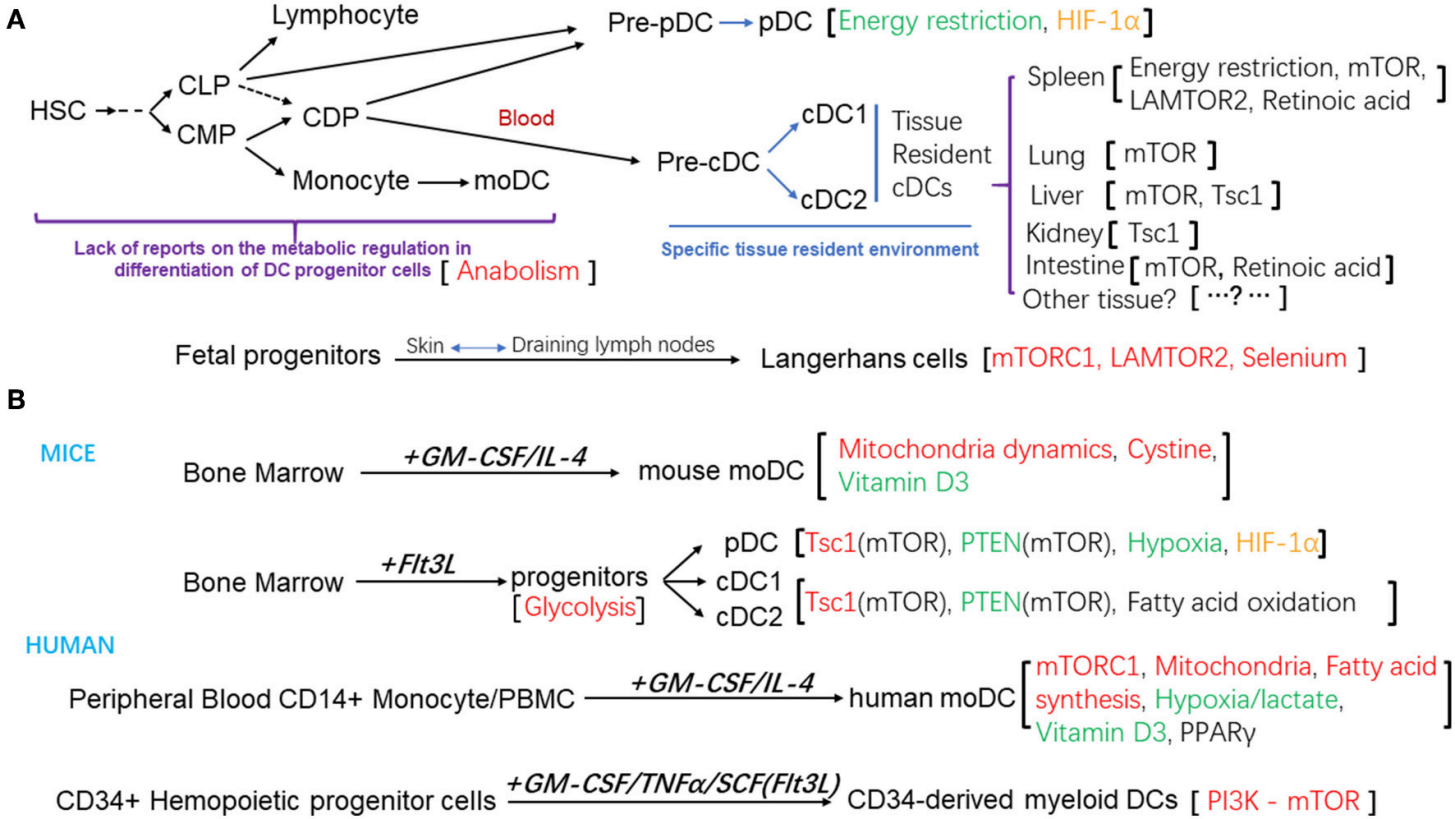
**(A)** Schematic diagram of the differentiation and development of DC subsets and the metabolic regulation factors that modulate these processes. **(B)** Different *in vitro* culture system for the generation of DCs from mouse bone marrow progenitors, or human peripheral blood mononuclear cells, CD14+ monocytes, or CD34+ Hemopoietic progenitor cells. The metabolic regulation factors were also listed. Positive regulators were in the red color, negative regulators were in the green color, regulators that affected the homeostasis of DC subsets were in the black color, regulator that is controversial for its role were in the orange color.

Apart from Langerhans cells which were shown to have an embryonic origin, most DC subsets are derived from hematopoietic stem cells (HSCs) (12). A series of DC progenitors have been identified based on their surface expression of molecules of hematopoietic progenitors, such as CD117, CD135, and their differentiation potential *in vitro* and *in vivo*. In mouse, both common myeloid progenitors (CMPs) and common lymphoid progenitors (CLPs) can give rise to all the DC subsets (13, 14). The common DC progenitors (CDPs) are the committed precursors for both pDCs and cDCs (15, 16). Within the bone marrow, CDPs differentiate into pre-cDCs and pre-pDCs, and pre-pDCs further differentiate into pDCs. Both pDCs and pre-cDCs then migrate from the bone marrow to the lymphoid and non-lymphoid tissues, where pre-cDCs terminally differentiate to cDC1 and cDC2 subsets (17–19). Similarly, human granulocyte macrophage progenitors (GMP) can be divided into three sub-populations, based on their differentiation potential determined by clonal analysis at single cell level: one give rise to granulocyte, monocyte and DCs, defined as hGMDP, which is equivalent to murine CMP; one produce monocytes and DCs, defined as hMDP; the hCDP subpopulation can only differentiate into DC subsets, equivalent to murine CDP (20, 21). The differentiation capacity of these DC progenitors and their relationships help to define the lineage map of DC development. In *in vitro* culture system, murine pDC, cDC1, and cDC2 subsets can be generated from the bone marrow cells in the presence of fms-like tyrosine kinase 3 receptor ligand (Flt3L); bone marrow cells can also differentiate into CD11c^hi^ MHC-II^hi^ CD11b^+^ DCs in the presence of granulocyte–macrophage colony-stimulating factor (GM-CSF) and IL-4 (22, 23). Human monocyte-derived DC (moDCs) can be obtained from purified blood CD14^+^ monocyte or total peripheral blood mononuclear cells in the culture system supplemented with GM-CSF and IL-4 (24). And human myeloid DCs or Langerhans cells can also be generated from human CD34^+^ hematopoietic progeniter cells with different cytokines (25–28). As shown in Figure 1B.

As metabolism is the essential process in all cell types, the effects of metabolic pathways on immune cell differentiation and functions have recently attracted great attention (29–32). Although limited, increasing numbers of studies are now revealing the importance of metabolic pathways involved in the modulation of DC development and differentiation. In this review, we will summarize the findings from recent studies on the metabolic regulation of DC differentiation and discuss the three major aspects that impact the processes of DC development and differentiation: the changes in metabolic pathways, the molecular signaling pathways that modulate cell metabolism, and the effects of metabolites and nutrients. Aiming to draw attentions to this promising research field where the effects of metabolic process and their regulator mechanisms in DC differentiation need to be further investigated.

## Role of Glycolysis and Mitochondria Function

Glycolysis is one of the most important components in glucose metabolism which converts glucose into pyruvate in the cytoplasm. Pyruvate then either transforms into lactate as metabolite of anaerobic glycolysis in the cytoplasm or enters Krebs cycle in mitochondria. Regulation of glycolysis in immune cell development, differentiation and/or activation has been well-characterized in T cells (33), B cells (34, 35), and macrophages (36). Growing evidences have shown that function of glycolysis is essential for DC activation (31), but its role during DC differentiation is less well-investigated. Recently Kratchmarov et al. showed that blockage of glycolysis by 2-deoxyglucose (2-DG) *in vitro* led to defects in Flt3L-induced mouse DC progenitor proliferation, indicating that glycolysis is required for DC development (37).

Under hypoxia condition, the conversion of pyruvate into lactic acid is favored, and ATP is generated for cellular energy supply. It was reported that lactic acid accumulated in DC cultures with high cell density induced reprogramming of human moDC differentiation, which vanish their ability to produce inflammatory cytokines and chemokines upon activation compared with moDCs developing at low cell culture density, instead they tend to produce the anti-inflammatory cytokine IL-10 upon activation (38). Another study showed that hypoxia condition suppressed the generation of pDCs from bone marrow progenitor cells in Flt3L supplemented culture system, and knockout of HIF-1α in monocyte/DC progenitors (MDP) in LysM-cre HIF-1α^fl/fl^ mice can reverse the defects caused by hypoxia condition. Although not stressed by the authors, it is notable that the number of cDC1s (CD24^+^ cDCs) other than cDC2 (SIRPα^+^ cDCs) also reduced under hypoxia condition (39).

Under the condition when oxygen is sufficient, pyruvate enters Krebs cycles whose products participate in oxidative phosphorylation (OXPHOS) to generate ATPs in mitochondria. This process generates more ATPs but at a slower rate compared with glycolysis. Compared to their precursor monocytes, larger number of mitochondria, higher endogenous respiratory activity, increased activity of the mitochondrial marker enzyme citrate synthase and a robust ATP production were observed in *in vitro* generated human moDCs. Inhibition of complex I in electron transport chain (ETC) by Rotenone resulted in an impaired differentiation of human moDCs accompanied by glycolysis compensation and compromised ATP production (40). Moreover, elevated mtDNA copy number and a rapid increase of Peroxisome proliferator-activated receptor gamma coactivator 1-alpha (PGC-1α) followed by upregulation of Mitochondrial transcription factor A (TFAM) and Nuclear respiratory factor 1 (NRF-1) were also observed during *in vitro* generation of human moDC (41). It is also reported that during GM-CSF induced bone marrow-derived mouse moDC generation, upregulation of mitochondrial fusion-related proteins was also observed, indicating an active mitochondrial dynamic during DC differentiation (42). These evidences implied an active state of mitochondria during DC differentiation. Recent systematic analysis on the differences of transcriptomics, proteomics and phosphoproteomics between CD8α^+^ DCs (cDC1) and CD8α^−^ DCs (cDC2) showed that CD8α^+^ DCs exhibit much stronger oxidative metabolism indicated by higher oxygen consumption rate (OCR). This indicated that aberrant mitochondria function may affect the expansion of CD8α^+^ DCs which hold great importance in CD8^+^ T cells mediated anti-tumor function *in vivo* (43).

Collectively, glycolysis is essential for the maintenance of DC progenitor cells, while proper function of mitochondria is required during the differentiation process of monocyte derived DCs both in human and in mouse. Researches described above all implied that that mitochondria function or oxidative metabolism, which produces more ATPs than anaerobic glycolysis in glucose metabolism, is favored in the development or expansion of the DCs subsets responsible for proinflammatory functions or antigen presentation to CD8^+^ T cells. As pDCs are the main source of Type I and Type III interferons among all DC subsets (Table 1) and cDC1s hold great importance in CD8^+^ T cells mediated anti-tumor function *in vivo*, these results may also point out that the hypoxia condition in the micro-environment of tumor mass may suppressed the expansion and function of the DC subsets which promote anti-tumor processes. Also avoiding hypoxia condition or inhibition of HIF1α in *in vitro* culture system would help to gain pDCs or cDC1s with proper functions for their clinical application more efficiently.

## Role of Fatty Acid Metabolism

Fatty acids can also serve as fuel for energy production in many types of cells (44). DC development from human PBMC precursors was diminished by blockade of fatty acid synthesis. *In vivo* experiments in mice suggest that dendropoiesis was also hampered after injection of fatty acid synthesis inhibitor to mice, as demonstrated by reduced CD11c^+^ cell numbers in liver, primary and secondary lymphoid organs (45). The nuclear receptor peroxisome proliferator activated receptor-γ (PPARγ), which is important in fatty acid metabolism is significantly up regulated in human monocyte derived DCs induced by GM-CSF and IL-4 *in vitro* and plays important role in human moDC generation (46–48).

Intriguingly, a recent report on the regulation of mTOR on metabolic adaption of DCs during allergic inflammation in lung using CD11c-cre mTOR^fl/fl^ mice indicated that the fatty acid metabolism, especially the fatty acid oxidation played important roles in the function and the expansion of inflammatory CD11b^+^ DCs in lung upon HDM induced allergic inflammation (49). This *in vivo* data indicated that the fatty acid oxidation that may be mediated by mTOR is essential for the generation of inflammatory DCs.

Moreover, Kratchmarov et al. reported that inhibition of catabolism-associated fatty acid oxidation with an inhibitor etomoxir did not affect the development of total cDCs and pDCs, but led to significantly increased frequency of IRF4 dependent cDC2 and decreased frequency of IRF8 dependent cDC1 cells in Flt3L-supplemented culture system (37). The homeostasis of changes of cDC subsets and their distinct functional features dependent on specialized signaling pathways and transcription factors (Table 1). This study implied that different cDC subsets may prefer specific metabolic status for their distinct functions, and the metabolic pathways may crosstalk with these signaling pathways and affect the differentiation of certain DC subsets. Crosstalk between fatty acid metabolism and glucose metabolism can be bridged by NADPH and acetyl-CoA. Defects in fatty acid oxidation will lead to the aberrant level of acetyl-CoA which may in turn affect the Krebs cycles in mitochondria. Notably, increasing evidences indicate that high concentrations of etomoxir may have off-target effects on inhibiting adenine nucleotide translocase (ANT) and the electron transport chain (ETC) in macrophage and T cells (50). Concentration of etomoxir used by Kratchmarov et al. was relatively high and off-target effects may also exist. *In vivo* DC specific knockout of Cpt1a the target of etomoxir is needed to validate the observation *in vitro*. However, together with the *in vivo* systematic research that cDC1 exhibit higher oxidative metabolism (43), both researches demonstrated that proper mitochondria function and oxidative metabolism is essential for the expansion of cDC1s.

On the other hand, fatty acid metabolisms such as the fatty acid synthesis and oxidations are frequently activated in tissue resident immune cells especially in liver and lung with distinct resident metabolic environments. The results of effect of fatty acid synthesis on the generation of moDCs and liver resident DCs, as well as fatty acid oxidation on the expansion of CD11b^+^ inflammatory DCs under allergic status in lung indicated that tissue resident environment maybe the major factor that affect the metabolism tendency during DC differentiation. Further studies on the effects of environmental factors on the metabolic pathways favored for the differentiation of various tissue resident DCs should provide new insights into how the differentiation of tissue resident DC is regulated by metabolism, and potential targets maybe identified for modulating these processes in certain disease settings.

## Role of Mammalian Target of Rapamycin (mTOR)

The mTOR pathway responds to various environmental cues such as nutrients and growth factors and controls numerous cellular processes that related to cell growth and metabolism. The mTOR protein is a serine/threonine protein kinase in the PI3K-related kinase (PIKK) family and mainly forms two functional protein complexes, mTORC1 and mTORC2 (51). In recent years growing evidence has shown that the mTOR pathway, especially mTORC1, plays essential role in the development and differentiation of DCs (52). Deletion of mTOR using *CD11c-Cre* system disturbed the homeostasis of tissue resident DC subsets in lung, spleen, liver as well as white adipose tissue and large intestinal lamina propria etc. (49). mTORC1 controls terminal myeloid differentiation by affecting population of the mature circulating monocytes and the development of neutrophils and DCs trough mTORC1-Myc pathway (53), and is essential for the development of cDCs, pDCs as well as Langerhans cells (52–58). In DC culture system that generate DCs for human hematopoietic precursors, the PI3K-AKT-mTOR pathway was stimulated during the GM-CSF and IL-4 induced monocyte-derived DCs differentiation. Inhibition of mTORC1 with rapamycin disrupted the GM-CSF signaling pathway and induced apoptosis of human moDCs in *in vitro* differentiation system (54, 55). The PI3K-mTOR pathway is also required for generation of pDCs and myeloid DCs from human CD34^+^ hematopoietic progenitor cells in *in vitro* culture system supplemented with different cytokines (56, 57). In mice, rapamycin which inhibits the mTOR pathway was shown to block the Flt3L induced generation of all DC subsets both in culture and *in vivo* (58, 59). And the homeostasis of Langerhans cells was proved to be depend on the mTORC1 pathways other than the mTORC2 pathways (60).

Activation of PI3K-AKT-mTOR pathway by deleting an intrinsic inhibitor—phosphatase and tensin homolog (Pten), greatly accelerated DC development in Flt3L-supplemented bone marrow (BM) culture system and can partially restore the defects caused by the presentation of rapamycin. The DC-specific loss of Pten (*Cd11-Cre* system) resulted in cell-intrinsic expansion of CD8^+^ or CD103^+^ cDC1 *in vivo*. However, Pten deletion showed little effect on DCs generated in GM-CSF supplemented BM cultures, indicating a diverse regulatory mechanism of Pten and mTOR pathways in different DC progenitors (59). Ablation of the tuberous sclerosis 1 (Tsc1), another negative regulator of mTORC1, using *Tsc1*^*f*/*f*^*-ERCre* system, led to more rapid expansion of BMDCs and bigger cell size than that of control cells in GM-CSF supplemented BM cultures (61). However, almost at the same time Wang et al. reported that knockout of Tsc1 using Rosa26-Cre-ER^T2^ system up-regulated cell metabolic programs including glycolysis, mitochondrial respiration and lipid synthesis, but significantly impaired DC development *in vivo* and in Flt3L-supplemented culture system. The mechanistic study revealed that a Tsc1-mTOR and Myc axis orchestrated metabolic programming during DC development (62). Myc, a critical transcription factor for stem cell and cancer cell proliferation was demonstrated to be one of the downstream effectors of mTORC1 (62). One of the paralogues of Myc, L-Myc was specially upregulated in DC progenitors and affect cDC subsets especially cDC1 in lung and liver, and it can be regulated by GM-CSF and IRF8. Furthermore, overexpression of c-Myc in Flt3^+^ CMPs reduced the proportion of mature cDCs and pDCs in Flt3L supplemented cultures (63). Although discrepancies exist in the regulatory mechanism of Pten and Tsc1, these results all suggest that the mTOR-Myc pathway is important for proliferation of DC progenitors and for expansion of DC subsets.

Discrepancies of results from studies described above implied a complicated and precise regulatory mechanism of mTOR pathway in the development and differentiation of DCs. On one hand, downstream effectors of Flt3L activated signaling pathway that mainly rely on activation of STAT3 and Flt3L supplemented mouse BM culture supports the generation cDCs and pDCs, while downstream effectors of GM-CSF activated pathway that mainly activates STAT5 which supports the generation of monocyte derived DCs, but suppress the pDC development from mouse BM (64, 65). It is still not clear how activation of PI3K-mTOR may cross talk with JAK-STATs pathways. Pten or Tsc1 may be involved differently in the activation of STAT3 or STAT5. On the other hand, deletion of Pten or Tsc1 may also activate other signaling or metabolic pathways that may be differently involved in the downstream of Flt3L or GM-CSF activated pathway, but more evidences are required to elucidate these possibilities.

Attenuating mTORC1 pathway by depleting Raptor, an essential component of mTORC1 pathway, in DCs resulted in expansion in splenic CD8^+^ cDCs and intestinal CD11c^+^CD11b^+^ cDCs (66). Another mTOR positive regulator, the late endosomal/lysosomal adaptor and MAPK and mTOR activator 2 (LAMTOR2) is a member of the Regulator/LAMTOR complex and regulates mTOR and extracellular signaling-regulated kinase (ERK) cascade. Deletion of LAMTOR2 in CD11c expressing cells (*Cd11c-Cre*) led to significant reduction of Langerhans cells in the epidermis soon after birth by impairing mTOR and ERK signaling (67). However, enlarged spleen and lymph nodes were observed with expanded cDCs and pDCs in aged mice with conditional knockout of LAMTOR2 in DCs (*Cd11c-Cre*). Since LAMTOR2 is also important for the endosome function, the accumulation of Flt3 on cell surface and downstream super-activated mTOR signal in LAMTOR2 knockout aged mice may be caused by a feedback regulation (68). Since LAMTOR2 has functions other than modulating the mTOR pathway, the consequences caused by LAMTOR2 knockout might be different in different DC subsets and a feedback regulatory mechanism may also contribute to the different outcomes in aged LAMTOR2 knockout mice.

On balance, these results all point out that precise regulatory network of mTOR is essential in DC development and differentiation. Although it was observed by Sinclair et al. that mTOR modulates the homeostasis of DC subsets in different tissues in quite diverse manners (49), the regulatory mechanisms of mTOR and the metabolic changes in various tissue resident DC differentiation warrant further investigation.

## Role of Nutrients

### General Effect of Nourishment

Energy restriction (ER) which is also known as calorie restriction was shown to inhibit the mTOR pathway. However, ER induces cell metabolic changes not only through inhibition of mTOR, but also through its principal upstream regulators—AMPK and Akt and its downstream targets p70S6K and 4E-BP1 (69, 70). NIH-31 is a rat and mouse diet standard set up by the National Institutes of Health that takes the nutrient loss during autoclaved sterilization in account. Comparing to mice that consumed NIH-31 diet *ad libitum* (have free access to food or water), ER mice that consumed 40% energy-restricted NIH-31 diet had significantly reduced bone marrow CDP, pre-DC populations and splenic CD8^+^ cDC and pDC populations (71).

### Vitamins

Vitamin A and vitamin D3 were shown to have crucial impact on DC differentiation.

By feeding mice with vitamin A-deficient diet or high vitamin A diet, Beijer et al. demonstrated that Vitamin A was specifically necessary for the development of RelB^high^ Notch-dependent CD4^+^, and CD8^−^CD4^−^ cDCs (72). Meanwhile, Klebanoff et al. reported that pan-retinoic acid receptors (pan-RARs) antagonist treatment caused a selective loss of the splenic ESAM^high^ cDC2 population and the developmentally-related intestinal CD11b^+^CD103^+^ cDCs (73). In addition to the terminal differentiation of cDC2, retinoic acid signaling was also shown to modulate the generation of gut-tropic migratory DC precursors—pre-mucosal DCs (pre-μDCs) from bone marrow progenitors both *in vitro* and *in vivo* (74).

1,25-Dihydroxyvitamin D3 (calcitriol) is the active form of vitamin D3. In 2000, four groups reported the inhibitory role of calcitriol in DC differentiation from murine or human monocyte *in vitro*. Addition of calcitriol impeded human and murine moDC differentiation from human PBMC or monocyte and murine bone marrow cells, respectively (75–78). While vitamin D receptor, the nuclear hormone receptor for vitamin D3, is repressed by IL-4 induced GATA-1 during human moDC differentiation, its expression is induced by TGF-β1 and has positive impact during human Langerhans cell lineage commitment (79). However, the cell-specific influence of VD3 on DC differentiation *in vivo* has not been properly addressed yet.

### Amino Acids

Amino acids as important components of proteins also take part in many metabolic processes. Glutathione was reported to play a crucial role in protecting cell from oxidative stress and it also has a protective role for DCs. The cystine/glutamate antiporter transports cystine (oxidized form of cysteine) into the cell for the glutathione biosynthetic pathway in exchange for glutamate. D'Angelo et al. reported that blocking cystine/glutamate antiporter activity impeded human moDC differentiation but did not affect LPS-induced DC maturation (80). The roles of other amino acids in DC differentiation are yet to be uncovered.

### Dietary Minerals

Selenium (Se) is an essential micronutrient that is important for metabolism process like proper thyroid hormone metabolism and has non-negligible effects on the immune system through its incorporation into selenoproteins. Inadequate intake of Se has been reported to compromise immune responses in animals and in human (81). Five weeks of Se-deficient diet treatment can decrease the epidermal Langerhans cell numbers by half in mice (82). The role of Se during DC differentiation has also been studied in chicken. Addition of inorganic Se (sodium selenite) in the culture system was reported to accelerate the differentiation of chicken DCs from chicken peripheral blood monocytes (83).

Studies above pointed out diverse effect of vitamins, amino acids and selenium on DC development. Other nutrients were also reported to have important effect on function or survival of DC. For example, vitamin C and vitamin E inhibits activation of human moDC upon proinflammatory cytokine stimulation (84); Zn^2+^ triggers murine moDC apoptosis through stimulating ceramide formation (85). Whether other nutrients influence differentiation of DCs awaits further study. Distinct tissue resident DC subsets with different functions are regulated by different tissue environments. Metabolic environment in different tissues may significantly impact the differentiation of pre-DCs to resident DC subsets. For example, in lung the cells have better access to oxygen in adipose tissue the fatty acid metabolism is more active, whereas in intestine, the metabolism of various carbohydrates, peptides and small nutrients are highly active. Intestinal DCs, for instance, are among the first line of immune cells that encounter dietary nutrients, thus, it is highly possible that these nutrients function as major regulators in the differentiation of intestinal DC from pre-DCs. Based on the finding that retinoic acid was involved in regulation of intestinal DC differentiation (73, 74), as well as the study showing that gut microbiota-derived short chain fatty acids could serve as competitive regulators for intestinal DC differentiation (86, 87), it is reasonable to assume that homeostasis of tissue resident DC subsets may also be susceptible to distinct metabolic pathways in other tissues. Further exploration of the exact roles of different metabolites and nutrients in the differentiation of different tissue resident DCs should provide new knowledge for better understanding the importance of metabolic regulation of DC differentiation and function, and the potential correlations between immune alterations and some metabolic diseases.

## Discussion and Conclusion

In recent years, emerging evidence has revealed that the metabolic modulation is essential for the development and function of immune system. Some evidence also suggested that the differentiation and activation of DCs might also be under metabolic modulation. A better understanding of the metabolic regulation of DC development and differentiation will not only help to establish the crucial network amongst various molecular regulatory mechanisms and metabolic regulations, but also help to elucidate the potential association of altered DC differentiation and activation with some metabolic diseases. However, current knowledge in this field is still limited. In this review we summarized these findings from published studies as shown in Figure 1. As reviewed by O'Neill et al. multiple metabolic inhibitors have been used in studies to validate the role of specific metabolic pathways in immune system (88). However, only few inhibitors were tested to determine the roles of metabolic regulation in DC differentiation. Most of the published studies were done with the *in vitro* culture systems supplemented with Flt3L or GM-CSF, although they provided useful information for these mentioned metabolic pathways in DC differentiation, clear and definitive conclusions can only be drawn from properly designed *in vivo* studies, and those should be the major focus of the further studies. Furthermore, little is known about the regulatory mechanisms of these metabolic pathways and their interplay/cross talk with other molecular or epigenetic regulation pathways known important for DC differentiation, such as regulations by transcription factors, cytokines and microRNAs. The impacts of other metabolic pathways including the pentose phosphate pathway (PPP) and nitrogen metabolism pathways on DC differentiation are yet to be determined. In addition, apart from the mTOR pathway, the effects of other molecular signaling pathways that regulate metabolism such as AMPK pathway on DC differentiation are not yet clearly elucidated. The role of other nutrients including minerals in DC differentiation also needs more attention for their easy access in daily diets.

Immunotherapy has shown a bright future for cancer treatment. The functions of DCs are crucial for the effectiveness of these therapies. Impairment of DC homeostasis or function are related to many diseases, such as inflammatory diseases (89, 90), autoimmune diseases (91) and cancer (92–94). The DC vaccines also hold a promising potential for developing more effective approaches for the treatment of various immune related diseases. The *in vitro* generation of various DC subsets from hematopoietic progenitor cells is non-substitutable in the studies of human DC differentiation. They can also serve as the main source of DCs for DC related therapies or DC vaccines. Modulation of specific metabolic pathways or addition of particular nutrients during the generation of DCs according to their metabolism requirements, may help to obtain specific DC subsets desired for various clinical applications. More extensive studies of the metabolic regulation of DC development and differentiation should be one of the priorities in the field of DC biology and the new knowledge gained from these studies will facilitate the clinical applications of DCs in the treatment of some immune-related diseases.

## Author Contributions

LW supervised the writing, analyzed, and edited this manuscript. ZH wrote, organized and edited the manuscript. XZ, ZS, and TW wrote part of the review.

### Conflict of Interest Statement

The authors declare that the research was conducted in the absence of any commercial or financial relationships that could be construed as a potential conflict of interest.
